# A Stretchable, Self-Healable Triboelectric Nanogenerator as Electronic Skin for Energy Harvesting and Tactile Sensing

**DOI:** 10.3390/ma14071689

**Published:** 2021-03-30

**Authors:** Xi Han, Dongjie Jiang, Xuecheng Qu, Yuan Bai, Yu Cao, Ruizeng Luo, Zhou Li

**Affiliations:** 1 School of Chemistry and Chemical Engineering, Center on Nanoenergy Research, School of Physical Science and Technology, Guangxi University, Nanning 530000, China; hanxi@binn.cas.cn (X.H.); baiyuan@st.gxu.edu.cn (Y.B.); caoyu@binn.cas.cn (Y.C.); 13026185974@163.com (R.L.); 2 CAS Center for Excellence in Nanoscience, Beijing Key Laboratory of Micro-Nano Energy and Sensor, Beijing Institute of Nanoenergy and Nanosystems, Chinese Academy of Sciences, Beijing 101400, China; jiangdongjie@binn.cas.cn (D.J.); quxuecheng@binn.cas.cn (X.Q.); 3 School of Nanoscience and Technology, University of Chinese Academy of Sciences, Beijing 101400, China

**Keywords:** stretchable, self-healable, hydrogels, triboelelectric nanogenerator, e-skin

## Abstract

Electronic skin that is deformable, self-healable, and self-powered has high competitiveness for next-generation energy/sense/robotic applications. Herein, we fabricated a stretchable, self-healable triboelectric nanogenerator (SH-TENG) as electronic skin for energy harvesting and tactile sensing. The elongation of SH-TENG can achieve 800% (uniaxial strain) and the SH-TENG can self-heal within 2.5 min. The SH-TENG is based on the single-electrode mode, which is constructed from ion hydrogels with an area of 2 cm × 3 cm, the output of short-circuit transferred charge (*Qsc*), open-circuit voltage (*Voc*), and short-circuit current (*Isc*) reaches ~6 nC, ~22 V, and ~400 nA, and the corresponding output power density is ~2.9 μW × cm^−2^ when the matching resistance was ~140 MΩ. As a biomechanical energy harvesting device, the SH-TENG also can drive red light-emitting diodes (LEDs) bulbs. Meanwhile, SH-TENG has shown good sensitivity to low-frequency human touch and can be used as an artificial electronic skin for touch/pressure sensing. This work provides a suitable candidate for the material selection of the hydrogel-based self-powered electronic skin.

## 1. Introduction

Flexible and wearable electronic devices have attracted much attention because of their potential applications in human health monitoring, intelligent sensing, and human–computer interaction systems [1,2]. At the same time, higher and higher requirements are placed on materials. Among them, stretchable and self-healable conductive materials have become a rapidly developing hot spot [3]. In recent years, stretchable, self-healable conductive hydrogels have attracted widespread attention in wearable electronics and energy harvesting applications, including smart healthcare devices, electronic skins, stretchable electrodes, flexible sensors, and other wearable electronics [4,5,6,7,8,9,10]. Gelatin is a product of collagen hydrolysis and contains a large number of functional groups. As one of the raw materials for preparing natural hydrogels, it is prone to cross-linking reactions [11]. Gelatin is easily soluble in water at 37 °C. Under low temperature conditions, gelatin molecular chains begin to form a triple helix structure to further form a three-dimensional network hydrogel, which has a reversible sol-gel transition characteristic. Meanwhile, gelatin has good biocompatibility and degradability [12,13], and does not cause immune response in the human body. These excellent characteristics make it have a good application prospect in the field of biomedicine. However, the mechanical properties of pure gelatin hydrogels are poor, and its wide range of applications can be achieved by improving the mechanical properties of gelatin-based hydrogels.

To adapt to the various arbitrary shapes where these flexible devices may appear, a shape-adaptable power supply is proposed to drive these flexible electronic devices [14,15,16]. Some existing flexible power sources such as lithium-ion batteries, electromagnetic generators, thermoelectric generators, solar cells, and nanogenerators have been widely used [17,18,19,20,21]. Among them, triboelectric nanogenerators (TENGs), as a green and sustainable energy source, are showing great advantages in the collection of low-frequency mechanical forces and are due to their wide range of material sources, diverse designability, low cost, and ease of manufacturing. TENGs have received widespread attention [16,22,23,24,25]. So far, after good structural innovation and material preparation, TENGs with good biocompatibility and shape adaptability have been rapidly developed [26,27,28,29]. When two materials with different electronegativity are frictionally contacted, charge transfer occurs between two materials, a potential difference is formed, in the external circuit, electrons are driven by the potential difference to flow between the two electrodes attached to the back of the triboelectric material layer or between the electrode and the ground to balance this potential difference. TENGs can effectively transform mechanical energy to generate electrical output, based on the triboelectric effect and electrostatic induction [23,26,30,31,32].

At present, many polymer materials have been applied in TENGs, and they have played a vital role in the friction layer and support materials of TENGs [33,34]. As a flexible, stretchable, and biocompatible functional material, the hydrogel constructed from hydrophilic polymer provides a suitable candidate for the design of flexible functional sensing electronic skin. However, when the electrode layer is damaged or cracked, it will cause fatal damage to the performance of the TENG [35]. Hence, it is essential to develop the self-healable and stretchable TENG. As a novel material, self-healable conductive hydrogels (SCHs) provide the preferred strategy to solve the above problems [36,37,38]. SCH is a semi-solid ionic conductor essentially. Its excellent self-healable performance, great mechanical, and conductive stability after deformation are of great significance to developing flexible electronics [39,40]. Harnessing hydrogel elastomers to wearable electronics has important significance and application prospects [41].

Herein, we prepared a flexible and self-healable single electrode TENG, achieved by introducing gelatin to polyacrylicacid (PAA), and add a specific molar concentration of NaCl as a conductive component. The prepared self-Healable triboelectric nanogenerator( SH-TENG) could achieve rapid self-healing in 2.5 min at room temperature. The SH-TENG could achieve super stretchability (~800% strain). Simultaneously, through the design of 8-pixel array, the sensor array is prepared to simulate electronic skin. It can be in conformal contact with the skin surface. The sensor array based on this design can generate an output voltage of ~10 V under a light touch. The electronic skin prepared with this SH-TENG can accurately realize simple human–computer interaction. The TENG constructed from the ionic hydrogel can also be used as an energy harvesting device, the generated electricity can directly light-emitting diode (LEDs). This work provides a new solution for the design of stretchable self-healable wearable electronic devices. 

## 2. Materials and Methods

### 2.1. Materials

Acrylic (AA, 99%) was purchased from Shanghai Yi ’en Chemical Technology Co., Ltd., (Shanghai, China). N,N′-methylene diacrylamide (MBA, ≥99.0%) was obtained from Shanghai Aladdin Biochemical Technology Co., Ltd., (Shanghai, China). Ammonium persulfate (APS), gelatin, and NaCl purchased from Sigma-Aldrich Co., Ltd. (Shanghai, China). Commercial very high bond (VHB) double-sided tape (3M VHB 9469) for encapsulation, purchased from 3M Co., Ltd. (Shenzhen, China). Ultra-pure water was used throughout the experiment.

### 2.2. Preparation of SCHs

The preparation process of SCH is shown below. SCH is synthesized by a simple “one-pot method.” Specifically, (1) 4.675 g NaCl was dissolved in 20 mL deionized water (the concentration of NaCl is 4 mol·L^−1^) under continuous magnetic stirring for 30 min at a rotation speed of 500 r·min^−1^ until the NaCl was utterly dissolved. (2) Simultaneously, add 6.0 g AA, 0.003 g MBA to the above clear solution, continue to stir for 30 min, and then add different amounts of gelatin (respectively 1.0 wt %, 2.0 wt %, 4.0 wt %, 8.0 wt %) to the reaction mixture until a gel-like mixture was obtained. (3) Add 0.0712 g APS (1.0 wt % AA) to the above solution to initiate the reaction, after 30 min, then filtering the mixed solution with a 0.5 μm filtration, finally transferring the solution to a glass mold with an area of 10 cm × 10 cm, the thickness of 2 mm, place it in an oven at 55 °C for 2 h to form SCH (the preparation process in Supplementary Materials Figure S1). The hydrogels with different mass fractions of gelatin all use 4 mol/L NaCl unless otherwise mentioned. The final SCH is sealed with a silica gel bag together with a glass mold at −20 °C.

Different mass fractions of gelatin were recorded as: PAA-Gel-NaCl-1.0, PAA-Gel-NaCl-2.0, PAA-Gel-NaCl-4.0, PAA-Gel-NaCl-8.0, respectively. Here, PAA-Gel-NaCl-1.0 represents an ionized hydrogel with a gelatin concentration of 1%. As a control group, we used the same method to design a pure PAA-NaCl hydrogel.

### 2.3. Characterization

The stress-strain test of SCH was performed on the ESM303/Mark-10 system, and the ascent speed was set to 20 mm·min^−1^. The MCR92 rheometer (Anton Paar, Shanghai, China) was used to study the dynamic oscillation frequency sweep of the hydrogel in the frequency range of 0.1 to 100 rad·s^−1^. A linear motor (Matsuura, Zurich, Switzerland) is used as the mechanical energy input source, and the descend speed is set to 1.5 m/s. A programmable electrometer (Keithley, Beijing, China) and the oscilloscope (Tektronix, Beaverton, OR, USA) were used to test the output performance of SH-TENG. The linear motor was used to provide periodic external force applied to the SH-TENG.

## 3. Results and Discussion

**Figure 1:**
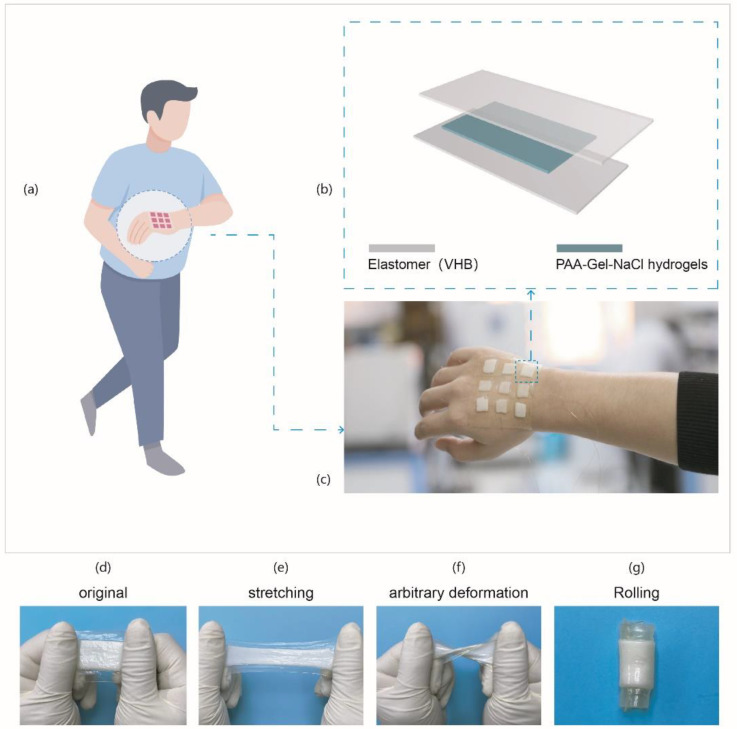
(**a**) Schematic diagram of electronic skin wearing model based on SH-TENG. (**b**) Scheme of the SH-TENG with sandwich structure. (**c**) An optical picture of an array SH-TENG patch attached to the back of the hand. (**d**) Original. (**e**) Stretching. (**f**) Arbitrary deformation. (**g**) Rolling.

Through a simple array arrangement, we designed a tactile artificial electronic skin. Figure 1a shows the electronic skin based on SH-TENG attached to the back of the curved hand. It is attached to human skin and connected with a metal wire. When the fingertips press different individual pixels on the sensor array, voltage signals output of different intensities will be generated. SH-TENG exhibits excellent tactile sensing performance and can be used as a self-power electronic skin for tactile sensing. SH-TENG is prepared with a classic sandwich structure as shown in Figure 1b. An enameled copper wire was fixed on the hydrogel with silver paste for electrical connection. For ease of operation, the thickness of the hydrogel and VHB tape are controlled at 2 mm and 85 μm, respectively. The final devices can be of arbitrary shapes. Figure 1c is a digital photo of the 8-pixel array SH-TENG patch prepared by the above method in conformal contact with the back of hand. As shown in optical images in Figure 1d, the prepared SH-TENG exhibits milk-white, it has excellent flexibility and will not damage the sensing function of the device under various mechanical deformations, such as stretching, knotting rolling, and so on (Figure 1e–g).

**Figure 2:**
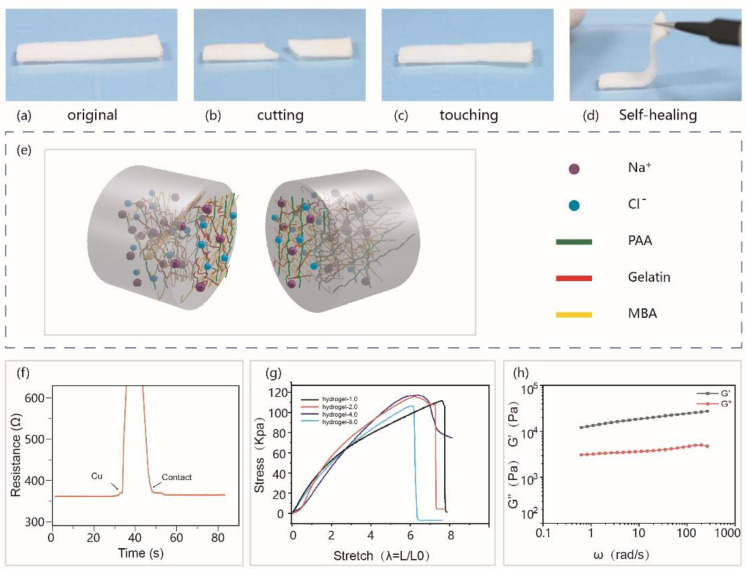
Self-healable property of PAA-Gel-NaCl hydrogel. (**a**–**d**) photographs of the PAA-Gel-NaCl hydrogel before and after self-healed. (**e**) The schematic diagram of the structure of the PAA-Gel-NaCl ionic hydrogels. (**f**) The capability of electrical recovery after sliced. (**g**) uniaxial tensile test of PAA-Gel-NaCl hydrogels with different gelatin content. (**h**) The storage and loss moduli as a function of frequency (1% strain).

The self-healable functional electronic skin is of great significance to the wearable flexible electronic devices. Figure 2 shows the self-healable result of PAA-Gel-NaCl hydrogel (Polyacrylic acid-Gelatin-Sodium chloride hydrogel). According to Figure 2a–d, the hydrogel was cut in half. Then, connected together in a record, the healing time, the hydrogel can self-heal in 2.5 min (The whole process is showed in movie S1, supporting information). Figure 2e shows the schematic diagram of the structure of the PAA-Gel-NaCl ionic hydrogels. The synthesized PAA-Gel-NaCl hydrogel can achieve rapid self-healing within 2.5 min at room temperature without any other stimuli. The excellent self-healing properties of the hydrogel may be due to the combined effect of the triple helix cross-linking of gelatin and the reversible cross-linking network formed by the dynamic hydrogen bond between PAA-Gel molecules, which helps to improve the mechanical properties of the hydrogel [42]. When the hydrogel is cut in half, the dynamic hydrogen bond between PAA molecules can be used as a special dynamic intersection point. Meanwhile, the reversible non-covalent coordination interaction of the PAA-NaCl hybrid network makes the ion hydrogel able to help rapid self-healing at room temperature. The capability of the electrical recovery after cut was demonstrated in Figure 2f, the resistance before slicing was 359.28 Ω. After 2.5 min of self-healing, the resistance returned to a stable 363.3 Ω, which was only a slight increase from the original state. Uniaxial tensile tests are performed to evaluate the mechanical properties of the hydrogel (Figure 2g). It can be seen from Figure 2g that doping with a small amount of gelatin increases the toughness of the hydrogel. When the mass fraction of gelatin is 1%, the strain of the hydrogel can reach 8 λ. When the mass fraction of gelatin reaches 8%, the breaking strength of the gel becomes 109.5 kpa, the strain of the hydrogel becomes 6.2 λ. Because the mechanical properties of gelatin are weak and brittle, too high gelatin concentration reduces Young’s modulus and breaking strength of the gel, and the brittle network of high concentration gelatin reduces the mechanical properties of the copolymer hydrogel. The frequency range of PAA-Gel-NaCl hydrogel under dynamic oscillation frequency sweep is from 0.1 to 100 rad·s^−1^, the relationship between storage energy (G′) modulus and loss modulus (G″) is shown in Figure 2h. Storage modulus is also the elastic modulus, which refers to the amount of energy stored due to elastic (reversible) deformation when the material is deformed, reflecting the elasticity of the material; Loss modulu is also the viscous modulus, which refers to the amount of energy lost due to viscous deformation (irreversible) when the material is deformed, reflecting the viscosity of the material. When the storage modulus and loss modulus are in an order of magnitude, the material is semi-solid, hydrogel material is a typical semi-solid substance. From the variation curve of the storage modulus (G′) and loss modulus (G″) of PAA-Gel-NaCl hydrogel with frequency (ω) (G′-ω curve, G″-ω curve), it can be seen that within the scanning range, the storage modulus of the hydrogel is always greater than the loss modulus at the dynamic oscillation frequency, indicating that the hydrogel exhibits stable elastomer-like properties in the frequency sweep range, and mainly exhibits elastic behavior.

**Figure 3:**
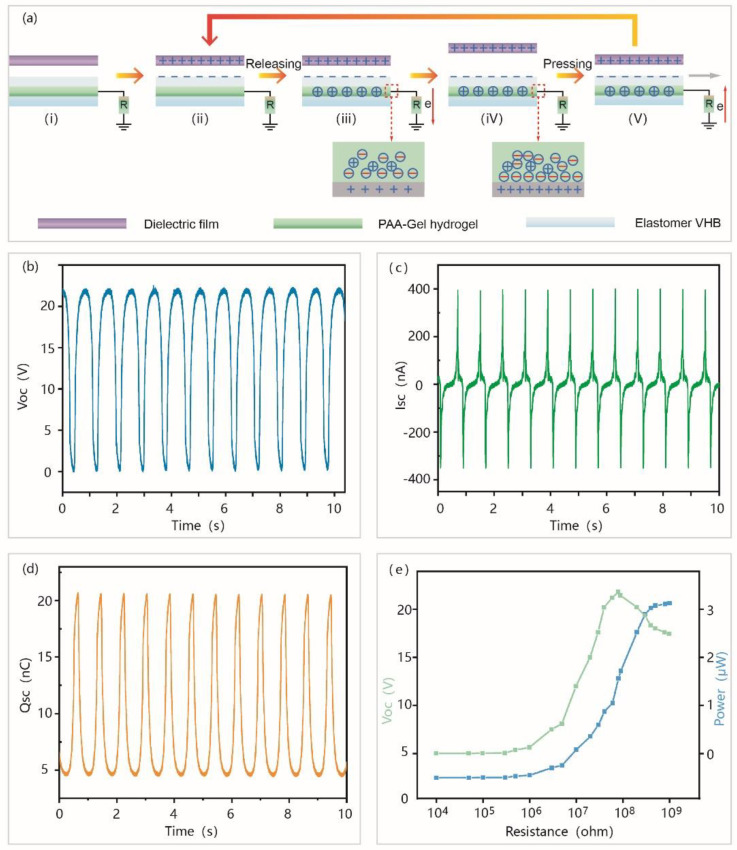
Schematic diagram of the SH-TENG in the single-electrode mode. (**a**) Schematic diagram of the working mechanism of the SH-TENG. (**b**–**d**) The measurements of the electrical output performances of the SH-TENG. (**b**) *Voc*. (**c**) *Isc*. (**d**) *Qsc*. (**e**) The output current density and power density change with the external resistance.

The PAA-Gel-NaCl hydrogel has good self-healable ability and mechanical properties. So, it is a suitable candidate material for preparing self-powered electronic skin based on TENG. TENG generally has four working modes: contact-separation mode, sliding mode, freestanding mode, and single-electrode mode [43]. The fundamental of the SH-TENG is the single-electrode mode (Figure 3a). To ensure the accuracy and repeatability of the experiment, the PTFE film (Poly tetra fluoroethylene) with high electronegativity was used to periodically contact with the VHB film of the friction layer of SH-TENG, the interface will be charged, and the same number of charges of opposite polarity will be generated on the surface of the dielectric film PTFE and VHB film, respectively (Figure 3(aii). During the periodic movement, when the two films are attached, there is no potential difference on the surface of the film because the two opposite charges overlap on the same plane. When the two surfaces are separated and moved away, the charge on the surface of the VHB film will induce ions to move in the hydrogel, thereby balancing the static charge and inducing opposite charges on the surface of the hydrogel (Figure 3(aiii). Simultaneously, an electric double layer is formed at the interface between the copper wire and the ion hydrogel, and the same number of negative ions are formed at the interface to induce positive ions that move in the direction of the copper wire (Figure 3(avi). In the double-layer structure, electrons flow from the metal wire to the ground through an external circuit until all the static charges in the electron beam are shielded by the elastic membrane (Figure 3(aiv). If the moving PTFE membrane approaches the elastomer membrane, the whole process will be reversed, and the reverse electron flux will be transferred from the ground to the interface between the copper wire and the ionized hydrogel (Figure 3(av). Through the regular contact and separation movement of the dielectric film PTFE and SH-TENG, alternating current will be generated.

When the PTFE film is in contact with SH-TENG, both *Voc* (open circuit voltage) and *Qsc* (the amount of charge transferred under open-circuit voltage) are zero. However, when the PTFE membrane and SH-TENG are gradually separated, the alternating current will be generated in the circuit. At this time, the relationship between *Voc* and *Qsc* conforms to the following formula:

(1) Voc=−σA/2C

(2) Qsc=−σA/2

In the above formula, *C* is the capacitance of the SH-TENG. *σ* is the density of electrostatic charge generated on the surface of the VHB film, and *A* is the contact area between the PTFE film and the VHB film.

We use a commercial PVDF film to perform the contact-separation motion (area, 2 cm × 3 cm) relative to SH-TENG. A linear motor with a frequency of 2 Hz and a speed of 0.5 m·s^−1^ was used to measure the electrical output of PP-TENG under contact-separation motion conditions. *Voc*, *Qsc*, *Isc* are shown in Figure 3b–d. It can be observed that SH-TENG can provide a stable output (*Voc* = 22 V, *Isc* = 400 nA, *Qsc* = 16 nC). The maximum power density of SH-TENG was ~2.9 μW·cm^−2^ when the matching resistance was ~140 MΩ (Figure 3e).

**Figure 4:**
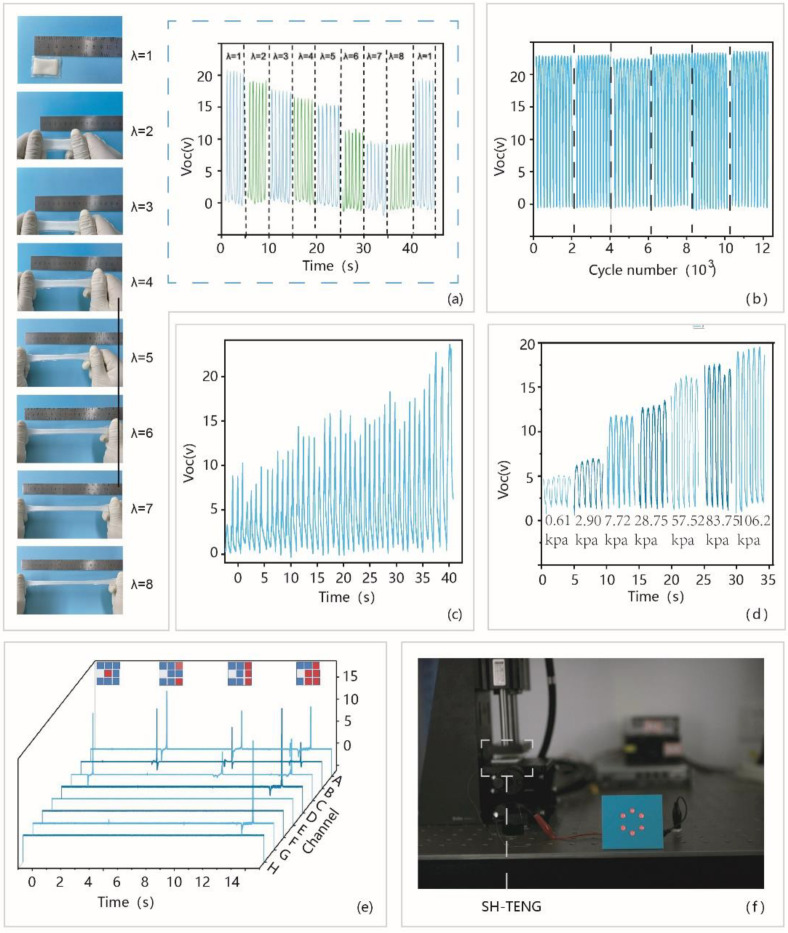
The stretchability and tactile sensing of the SH-TENG. (**a**) Digital photographs of an SH-TENG at initial state and different stretched states and *Voc* output of SH-STENG under different stretch rates. (**b**) *Voc* of the SH-TENG that lasted for ~12,000 cycles of contact-separation motions. (**c**) The voltage signal obtained by pressing the tactile sensor with an area of 2 cm × 3 cm under increasing pressure. (**d**) Detection range of the tactile sensor was quantified by using the commercial pressure sensor. (**e**) The voltage signal obtained by pressing the sensor array of different pixels with fingers. (**f**) An image of six red light-emitting diodes (LEDs) lightened by the SH-TENG.

The flexible SH-TENG has a linear output response under different gradient tensile strains. The feasibility of SH-TENG energy harvesting in the stretched state was further evaluated. The SH-TENG (2 cm × 3 cm) is uniaxially stretched to different stretches or strains (Figure 4a), when it comes into contact with a fixed shape and size dielectric film (VHB) during contact-separation movement, the corresponding electrical output is recorded. Compared with the initial unstrained state (λ = 1), when stretch λ = 2–8, the open circuit voltage of SH-TENG shows a decreasing trend, when λ = 8, the open circuit voltage only accounts for the initial unstrained half of the output in the state reaches ~11 V. The decrease in *Voc* is due to the reduction in the contacted surface area of the hydrogel, which reduces the charged contact area in the stretched state. After recovering from the stretched state, the electronic output is equivalent to the initial state, indicating that the device is not degraded. Figure 4b shows stability tests of SH-TENG. Generally, tactile sensor based on the SH-TENG is typically vulnerable to the external environment, so it is necessary to measure its long-term output performance and stability. After 12,000 working cycles, the open-circuit voltage of tactile sensor remained stable relative to the initial state, showing its excellent durability and stability. Tactile sensor exhibits good tactile sensing characteristics, and has demonstrated great applications in artificial electronic skin. As shown in Figure 4c, an area of 2 cm × 3 cm of the tactile sensor is prepared by using a sandwich structure and placed on the back of the hand. Under the linear increased touch pressure, the open-circuit voltage of the sensor also shows a linear increase. Similarly, the detection range of the tactile sensor was quantified by using the commercial pressure sensor. The sensor showed good sensing characteristics in the low-pressure range, with a minimum pressure detection limit of about 0.61 kpa. The voltages recorded under different contact pressures are shown in Figure 4d. It further shows that our device can achieve accurate pressure response, which can be used in a simple human–computer interaction system. Figure 4e shows the prepared 8 pixel sensor array. The number of the pixels are related to the number of unit devices in the array. An eight-channel oscilloscope was used to test the performance of the tactile sensing. By touching different pixels, the sensor array corresponds to the open-circuit voltage signal. This flexible array-type pixel strip shows a great potential application in human–computer interaction interface, when the pixels are dense enough, high-resolution pressure recognition can be performed. As a biomechanical energy harvesting device, SH-TENG can be used to collect low-frequency forces (Figure 4f). Six red light-emitting diodes (LEDs) were lightened by the SH-TENG. The working process is fully demonstrated in Video S2.

## 4. Conclusions

In summary, we prepared PAA-Gel-NaCl hydrogel by a simple in situ free radical polymerization method, and then prepared a stretchable, self-healable single electrode TENG based on the hydrogel. The SH-TENG can provide a stable output (*Voc* = 22 V, *Isc* = 400 nA, *Qsc* = 16 nC), the maximum output areal power density is ~2.9 μW·cm^−2^ at a matched resistance of ~140 MΩ. The electrical output of the SH-TENG is sensitive to pressure, and the voltage output also changes linearly under linear pressure changes. The electronic skin prepared by using this SH-TENG can accurately realize simple human–computer interaction applications, and it is expected to expand the use scene by increasing the pixel resolution. As a biomechanical energy harvesting device, it also can be used to collect low-frequency forces. We successfully light up a set of LEDs. In the next research, we can develop other flexible electrodes with greater deformability under tension and maintain the electrical output performance of flexible electrodes. We believe that TENG based on ionic hydrogel has advantages in flexible and wearable electronic devices.

## Data Availability

Data is contained within the article.

## Supplementary Materials

The following are available online at https://www.mdpi.com/article/10.3390/ma14071689/s1, Figure S1: Synthesis route and Formation mechanism of PAA-Gel-NaCl hydrogel. Video S1: Powering a set of LED lights by SH-TENG. Video S2: Self-healable video of PAA-Gel-NaCl hydrogel.
