# Supplementary material for: A Stretchable, Self-Healable Triboelectric Nanogenerator as Electronic Skin for Energy Harvesting and Tactile Sensing
Source: Materials (Basel). 2021 Mar 30;14(7):1689. doi: 10.3390/ma14071689 (PMC8036526; doi:10.3390/ma14071689)
Supplement: Supplementary file 1 [file materials-14-01689-s001.zip › materials-1147609 supplementary/Supplementary information.docx]

Article

A stretchable, self-healable triboelectric nanogenerator as electronic skin for energy harvesting and tactile sensing

Xi Han^# 1^, Dongjie Jiang^# 2,3^ ,Xuecheng Qu ^2,3^, Yuan Bai ^1^, Yu Cao ^1^, Ruizeng Luo ^1^ and Zhou Li ^1,2,3^*

^1^ School of Chemistry and Chemical Engineering, Center on Nanoenergy Research, School of Physical Science and Technology, Guangxi University,Nanning 530000,China; hanxi@binn.cas.cn (X.H.); baiyuan@st.gxu.edu.cn (Y.B.); caoyu@binn.cas.cn(Y.C.); 13026185974@163.com(R.Z.L.)

^2^ CAS Center for Excellence in Nanoscience, Beijing Key Laboratory of Micro-nano Energy and Sensor, Beijing Institute of Nanoenergy and Nanosystems, Chinese Academy of Sciences, Beijing, China; jiangdongjie@binn.cas.cn (D.J.J.); quxuecheng@binn.cas.cn(X.C.Q.)

^3^ School of Nanoscience and Technology, University of Chinese Academy of Sciences, Beijing, China;

***** Correspondence: zli@binn.cas.cn; Tel.: ＋86-138-1192-2339


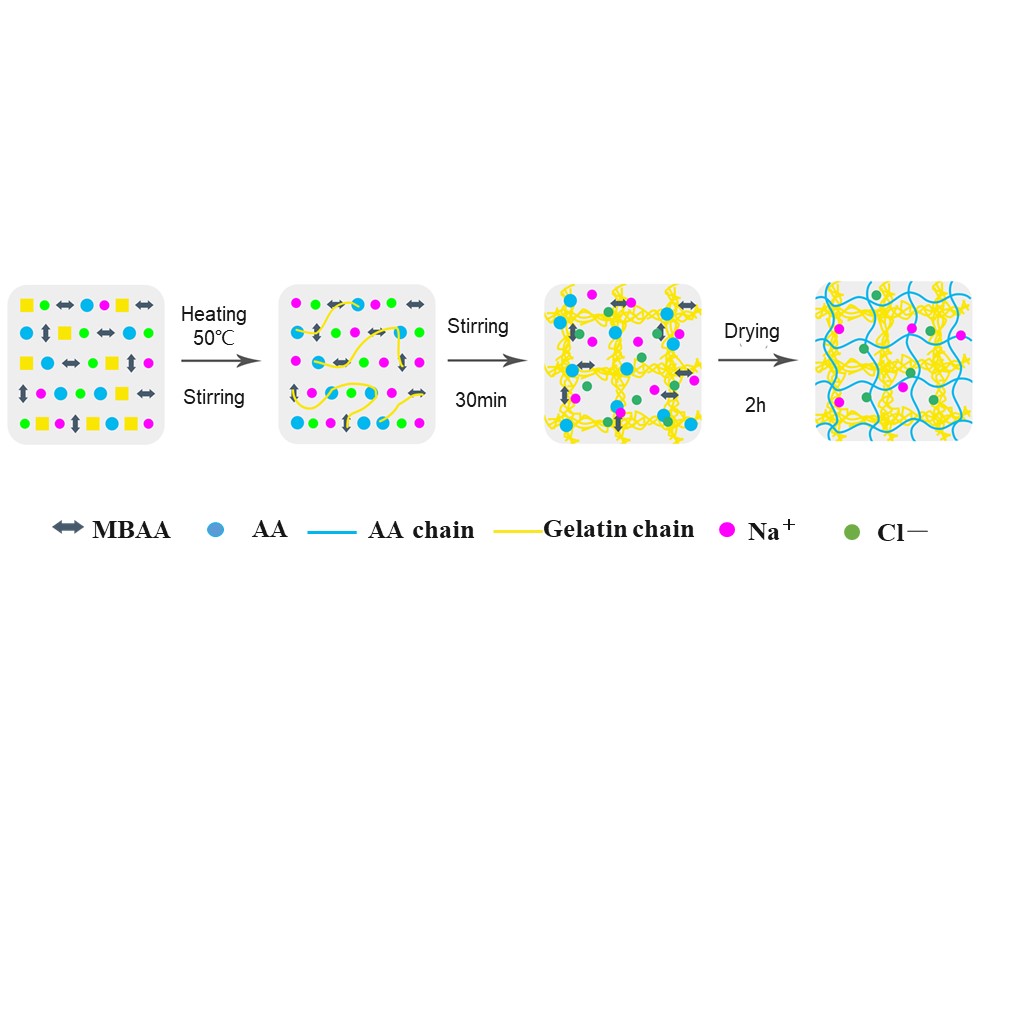


**Figure S1.** Synthesis route and Formation mechanism of PAA-Gel-NaCl hydrogel.

| **Citation:** Lastname, F.; Lastname, F.; Lastname, F. Title. *Materials* **2021**, *14*, x. https://doi.org/10.3390/xxxxx  Academic Editor: Firstname Lastname  Received: date  Accepted: date  Published: date  **Publisher’s Note:** MDPI stays neutral with regard to jurisdictional claims in published maps and institutional affiliations.  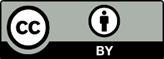  **Copyright:** © 2021 by the authors. Submitted for possible open access publication under the terms and conditions of the Creative Commons Attribution (CC BY) license (http://creativecommons.org/licenses/by/4.0/). |
| --- |

Specifically, (1) Prepare 20 ml of NaCl solution with a molar concentration of 4 mol L^-1^, and stir magnetically until the solution becomes clear. Add 6.0 g AA and 0.003 g MBA to the clear solution, continue stirring for 30 minutes, and then add gelatin with different mass concentrations to obtain hydrogels with different mass concentrations of gelatin. Add 0.0712g APS to the above solution to initiate the reaction. After 30 minutes, filter the mixed solution with a 0.5μm filtration. Finally, transferring the solution to a glass mold with an area of 10cm×10cm，the thickness of 2mm, place it in an oven at 55℃ for 2h to form the hydrogels.

References

1. Hussain, A.M.; Hussain, M.M. CMOS-Technology-Enabled Flexible and Stretchable Electronics for Internet of Everything Applications. *Advanced Materials* **2016**, *28*, 4219-4249, doi:10.1002/adma.201504236.

2. Lipomi, D.J. Stretchable Figures of Merit in Deformable Electronics. *Advanced Materials* **2016**, *28*, 4180-4183, doi:10.1002/adma.201504196.

3. Sekitani, T.; Zschieschang, U.; Klauk, H.; Someya, T. Flexible organic transistors and circuits with extreme bending stability. *Nature Materials* **2010**, *9*, 1015-1022, doi:10.1038/Nmat2896.

4. Someya, T.; Kaltenbrunner, M.; Yokota, T. Ultraflexible organic electronics. *Mrs Bull* **2015**, *40*, 1130-1137, doi:10.1557/mrs.2015.277.
